# Evaluating the generalisability of LiSep LSTM for early prediction of septic shock across US and european cohorts

**DOI:** 10.1038/s41598-026-63350-0

**Published:** 2026-09-10

**Authors:** Hong K. Tan, Logan Froese, Daniel Wilhelms, Josef Fagerström, Michelle. S. Chew

**Affiliations:** 1 Department of Anaesthesiology and Intensive Care Medicine, Linköping University Hospital, Linköping, 581 85 Sweden; 2 Department of Clinical Neuroscience, Karolinska Institutet, Stockholm, Sweden; 3 Department of Neurology, Karolinska University Hospital, Stockholm, Sweden; 4 Division of Drug Research, Department of Medical and Health Sciences, Faulty of Health Sciences, Linköping University, Linköping, 581 83 Sweden; 5 Department of Emergency Medicine, Local Health Care Services in Central Östergötland, Region Östergötland, Linköping, 581 91 Sweden; 6 Department of Computer and Information Science, Linköping University, Linköping, 581 83 Sweden; 7 Department of Perioeperative Medicine and Intensive Care, Karolinska University Hospital, Stockholm, Sweden

**Keywords:** Sepsis, Septic shock, LiSep LSTM, early prediction, generalisability, Diseases, Health care, Medical research

## Abstract

Sepsis remains a major public health concern and is associated with high mortality. Early detection and timely intervention are critical for improving outcomes, yet no standardised approach has been universally adopted. The LiSep LSTM model, a Long Short-Term Memory neural network developed using the MIMIC-III database, has demonstrated promising predictive performance but generalisability across regions has not yet been explored. This study evaluated the LiSep LSTM model in an independent European cohort from the AmsterdamUMC (AUMC) database. Model performance was assessed using internal, external, and pooled validation to examine the effects of distributional shift and data heterogeneity across cohorts. Permutation feature importance (PFI) was further applied to quantify each feature’s contribution to model performance across evaluation settings. When tested on the AUMC cohort, the model achieved an Area Under the Receiver Operating Characteristic Curve (AUROC) of 0.8851 (95% confidence interval [CI] 0.8612–0.909) with a median Hours Before Onset (HBO) of 47.0 hours (interquartile range [IQR]: 16.0–255.0), slightly outperforming its performance on MIMIC-III. External validation showed reduced calibration and recall, while pooled training improved AUROC and Brier score relative to external-only evaluation. Accuracy was moderate (0.60–0.77) and precision remained low (0.01–0.17), indicating a substantial false-positive burden. These findings suggest that pooled training enhances robustness and supports earlier identification of septic shock, but local calibration and strategies to manage false alarms remain necessary for reliable deployment.

**Supplementary Information:**

The online version contains supplementary material available at https://doi.org/10.1038/s41598-026-63350-0.

## Introduction

Sepsis represents a persistent challenge in critical care, with substantial associated mortality across intensive care units (ICUs) worldwide. According to the Sepsis-3 definition, sepsis is characterised as life-threatening organ dysfunction caused by a dysregulated host response to infection^1^. It can arise from bacterial, viral, fungal, or parasitic infections and affects patients of all ages^2^. Septic shock, the most severe complication, is defined by persistent hypotension requiring vasopressor therapy despite adequate fluid resuscitation and is associated with markedly increased mortality risk^1^. A recent meta-analysis reported average sepsis mortality rates of 24.4% at 30 days and 32.2% at 90 days, while septic shock mortality reached 34.7% and 38.5%, respectively. Moreover, international studies have demonstrated geographic variation, with septic shock mortality ranging from 26.4% in Australia to over 40% in Europe, underscoring the need for improved, generalisable clinical tools^3^.

Electronic health records (EHRs) have facilitated the development of machine learning (ML) approaches for early detection of sepsis and septic shock^4^. Several ML-based early warning systems have demonstrated promise^5^, with retrospective validation suggesting that timely prediction could improve clinical outcomes, as each hour of delayed antimicrobial therapy significantly worsens survival^6^. However, there is currently no universally accepted standard for early detection^2^, and most predictive models suffer from limited external validity. While multiple studies on early identification of septic shock have yielded promising result^5,7–10^, algorithms developed on single-centre or single-country dataset often fail to generalise across healthcare systems due to differences in patient demographics, clinical practices, and EHR structures.

Several multi-centre retrospective studies have explored the potential of ML for sepsis prediction. For example, the TREWScore model was prospectively deployed across five hospitals on the U.S. east coast, where it was used to investigate the association between sepsis alerts, timing of prediction, and subsequent antibiotic ordering^11,12^. In 2021, Weng et al. applied data from both the United States and China to predict mortality in ICU patients with sepsis using univariate logistic regression^13^. In 2022, Moor et al. combined four large ICU databases from the U.S., the Netherlands, and Switzerland to benchmark deep learning and traditional ML models against established early warning scores for early sepsis detection^14^. More recently, Zhang et al. assessed in-hospital mortality prediction among sepsis patients across U.S. and Dutch cohorts, evaluating seven different ML-based predictive models^15^.

The LiSep LSTM model, introduced in 2019, achieved state-of-the-art performance for early septic shock detection using the MIMIC-III database, a large U.S. based critical care dataset^16^. LiSep LSTM outperformed contemporary ML methods on both area under receiver operating curve (AUROC) and hours before onset (HBO) metrics, extending reliable prediction windows to nearly 20 HBO while maintaining strong discriminative ability^17^. Despite these promising results, LiSep LSTM was developed exclusively on U.S. ICU data, limiting its applicability to other clinical environments. Healthcare systems across continents differ in care pathways, patient characteristics, and documentation practices, raising concerns about cross-cohort robustness.

To address this gap, we evaluate LiSep LSTM across two large-scale ICU datasets from different continents: MIMIC-III (U.S.) and AUMC (Netherlands). These datasets enable a systematic assessment of cross cohort transferability. Specifically, we: (1) assess the internal performance of LiSep LSTM within each cohort, (2) conduct external validation across datasets, and (3) perform pooled training to examine whether combining data from both continents improves generalisation.

Model performance is reported using AUROC and HBO as the primary evaluation metrics, and further assessed by Brier score, accuracy, precision, and recall to characterise calibration, classification balance, and predictive reliability. We additionally analyse the cohort-level distributions of key septic shock features to characterise differences in patient populations and data structures between the two cohorts. Finally, permutation feature importance (PFI) is applied to quantify the contribution of individual variables to model prediction and to examine how feature relevance changes across internal, external, and pooled evaluation settings.

This study investigates the stability and transferability of the LiSep LSTM model for the early detection of septic shock. Our findings aim to provide insights into its potential for deployment in European healthcare systems, where patient demographics and clinical practices may differ from U.S. contexts.

## Methods

We conducted a retrospective study using two publicly available critical care EHR databases: MIMIC-III and AUMC. MIMIC-III contains approximately 59,000 ICU admissions from Beth Israel Deaconess Medical Center, Boston, USA, between 2001 and 2012^16^. The AUMC database includes roughly 23,000 ICU admissions from Amsterdam University Medical Centers, the Netherlands, spanning 2003–2016^18^. Both datasets are de-identified and provide comprehensive clinical information, including demographics, vital signs, laboratory results, medications, diagnoses, and observation notes. The data are distributed in comma-separated value (CSV) format and imported into a PostgreSQL database for preprocessing and feature extraction ^19^. MIMIC-III complies with HIPAA regulations, while AUMC adheres to both HIPAA and GDPR requirements. As both datasets are anonymised and publicly available, ethical approval was not required for the present study.

### Hardware configurations

LiSep LSTM model is implemented in Python using Keras framework with Google TensorFlow as backend ^20,21^. The hardware configuration used for ML model training as well as data pre-processing was NVIDIA GeForce RTX™ 4060 GPU 8 GB GDDR6, Intel Core i7-12700H Processor 2.3 GHz CPU with 16 GB DDR4 RAM.

### Definition of septic shock

#### Sepsis

Sepsis was defined according to the SIRS based criteria^22^ and TREWScore^7^ framework adopted in the original LiSep LSTM study^17^. Patients were identified as having SIRS if at least two SIRS criteria were present simultaneously. Suspicion of infection was defined using ICD-9 diagnosis codes indicating infection, consistent with the approach described by Angus et al.^23^, or by the presence of a clinical note mentioning sepsis. Patients meeting both SIRS and suspected infection criteria were subsequently classified as sepsis positive.

The SIRS criteria evaluated in this study were defined as follows:

- Heart rate (HR) > 90 beats per minute
- Body temperature > 38 $^\circ$ C or < 36 $^\circ$ C
- Respiratory rate (RR) > 20 breaths/min or Partial Pressure of Carbon Dioxide (PaCO_2_) in arterial blood < 32 mmHg
- White blood cell count > 12,000/mL or < 4,000/mL

#### Severe sepsis

Severe sepsis is defined as sepsis accompanied by organ dysfunction related to sepsis, following the Surviving Sepsis Campaign guidelines ^24^. Sepsis related organ dysfunction can be identified by any of the following symptoms:

- Lactate > 2 mmol/L
- Systolic blood pressure < 90 mm Hg
- Urine output < 0.5 mL*/*kg, over 2 hours despite adequate fluid resuscitation
- Creatinine > 2 mg/dL without renal insufficiency or chronic dialysis
- Bilirubin > 2 mg/dL without having liver disease or cirrhosis
- Platelet count < 100 000 µL
- International normalised ratio > 1.5
- Acute lung injury with arterial oxygen pressure (PaO_2_)/fractional inspired oxygen (FiO_2_) is less than 200 in the presence of pneumonia
- Acute lung injury with PaO_2_/FiO_2_ less than 250 without pneumonia.

#### Septic shock

Septic shock was defined by the presence of severe sepsis and hypotension systolic blood pressure < 90 mmHg despite adequate fluid resuscitation, where adequate resuscitation was considered as administration of ≥ 20 mL/kg within 24 hours or receipt of a total fluid volume ≥ 1200 ml.

Each admission was screened on an hourly basis from admission until ICU discharge or the end of the recorded stay. The first hour meeting criteria for sepsis, severe sepsis, or septic shock was defined as the septic shock onset time and used as the prediction target.

### Data preprocessing

Both MIMIC‑III and AUMC datasets provide event‑level clinical data recorded at irregular intervals, with vital signs often measured multiple times per hour and laboratory values sampled anywhere from hourly to once daily depending on clinical practice. All raw observations were therefore resampled to an hourly grid, which served as the fundamental temporal resolution for both feature construction and outcome labelling.

**Fig. 1:**
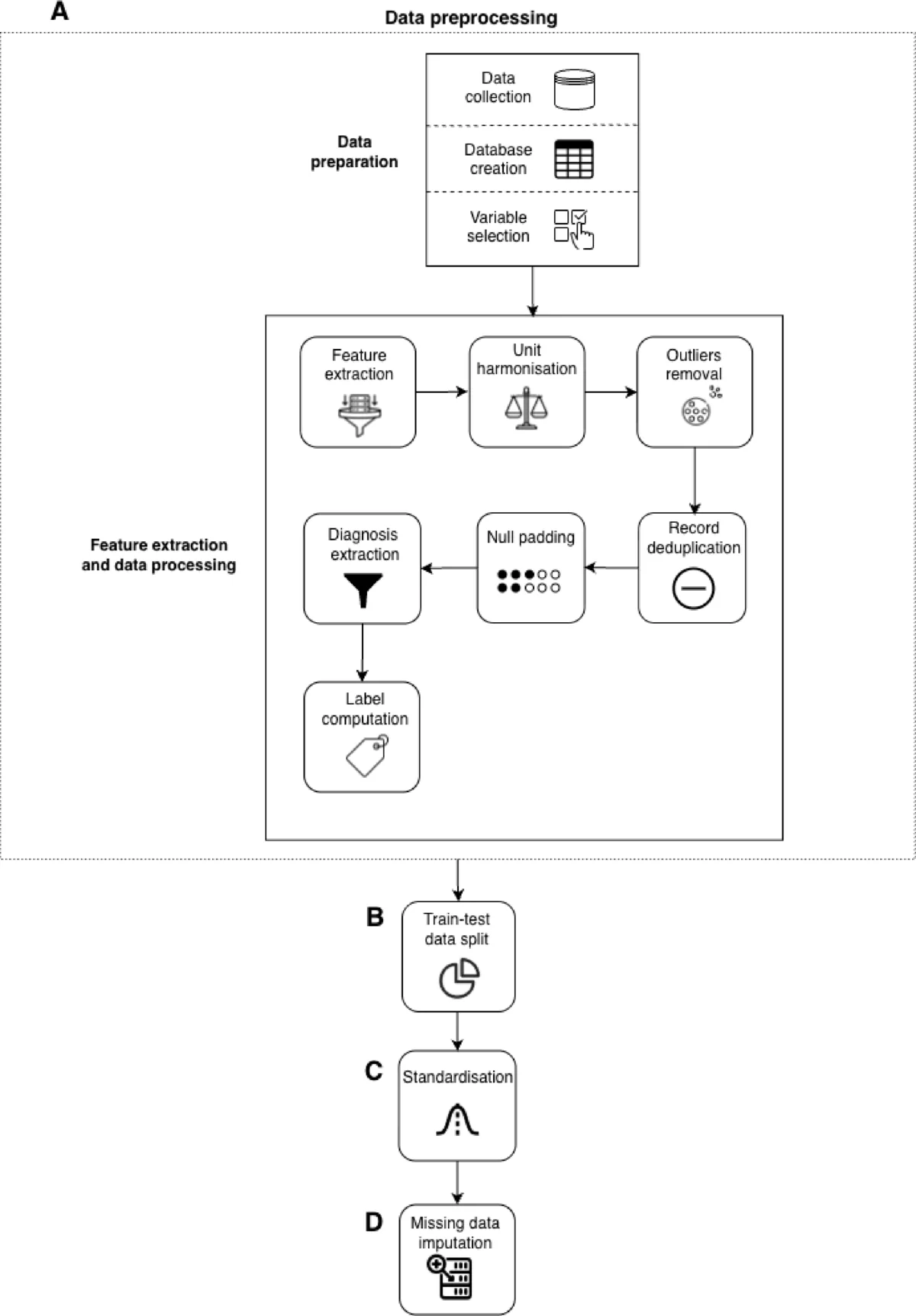
Overview of the preprocessing pipeline applied to MIMIC-III and AUMC prior to LiSep LSTM model development. Dataset partitioning was performed before feature standardisation and missing data imputation to prevent information leakage.

An overview of the preprocessing pipeline is shown in Fig. 1. All preprocessing steps as shown in Fig. 1A were designed to remain consistent with the procedures described in the original LiSep LSTM study by Fagerström et al. ^17^.

- *Data preparation*. Separate PostgreSQL databases were constructed for MIMIC-III and AUMC due to differences in table structure, variable naming, and data organisation between the two cohorts. Variables were selected to match those reported by Fagerström et al.^17^ (complete variable list provided in Supplementary Table 2). Patients younger than 15 years were excluded ^24^.
- *Feature extraction*. Time series features were extracted independently for each ICU admission. Each clinical measurement was linked to its elapsed time from ICU admission and assigned to an hourly interval relative to admission. For example, measurements recorded 20 and 50 min after admission were both assigned to Hour 1, whereas a measurement recorded 75 min after admission was assigned to Hour 2. This established a common hourly framework for subsequent preprocessing and temporal alignment.

Variables sampled less frequently than hourly were assigned to the corresponding hourly timestep based on their recorded timestamp. Measurements were therefore represented only at the timesteps at which observations occurred. For example, a measurement recorded 30 minutes after admission and again 24 hours later would be assigned to Hour 1 and Hour 24, respectively. This approach preserved the original measurement frequency while representing all variables within a common hourly framework.

- *Unit harmonisation*. Clinical variables were harmonised to consistent measurement units across MIMIC-III and AUMC by applying unit conversions where required, to align equivalent variables to a common scale prior to model development. Records that could not be harmonised to the expected measurement unit were subsequently removed.
- *Outlier removal*. Outlier detection was performed using predefined plausibility bounds to remove implausible or miscoded values (Supplementary Table 2). These thresholds were reviewed in consultation with clinical collaborators to exclude implausible or miscoded values while retaining clinically extreme but physiologically plausible observations.
- *Record deduplication*. Following feature extraction, multiple entries occasionally remained when multiple observations of the same variable for the same admission shared an identical timestamp. For physiological variables such as vital signs, multiple observations within the same timestep were aggregated using the mean value to obtain a single representative measurement while reducing the influence of minor recording discrepancies. For cumulative variables including fluid input, vasoactive medication administration, and urine output, values recorded within the same timestep were summed to preserve the total recorded quantity represented by multiple entries within the same timestep. Antibiotic administration records were retained as a single occurrence within each timestep. Due to heterogeneous measurement units, antibiotic treatment was represented as an administration event rather than a quantitative measurement. When multiple antibiotic administration records were present within the same timestep, they were counted as a single administration event to avoid repeated counting.

Each row represented a single admission at a specific hourly timestep, with all available physiological, laboratory, and treatment related variables aligned within a common temporal framework.

- *Null padding*. For each ICU admission, all variables were aligned to a continuous hourly sequential timeline spanning the full admission sequence. Missing hourly intervals between the first and the last recorded sequential hours were padded with null rows to preserve temporal continuity and ensure consistent sequence structure for subsequent time series modelling. Sequence lengths were allowed to vary across admissions.
- *Diagnosis extraction*. ICD-9 diagnosis codes were extracted and transformed into binary indicator flags representing the presence or absence of predefined clinical conditions for each ICU admission (Supplementary Table 2). These flags were generated once for each admission and subsequently linked to the corresponding admission records.
- *Label computation*. Outcome labels and onset times were generated according to the sepsis, severe sepsis, and septic shock definitions described earlier in the Methods section, consistent with the hourly annotation strategy used by Fagerström et al.^17^ . Labels were annotated at the admission level using a sequential rule-based labeling framework. Diagnostic criteria were evaluated chronologically across hourly aggregated patient sequences. To reflect clinical progression, the onset label for each stage was assigned at the earliest sequential hour satisfying its corresponding criteria. Sepsis onset was assigned at the first hourly timestep meeting the sepsis criteria described in “Definition of septic shock” section. Septic shock onset was then assigned at the first hourly timestep meeting the septic shock criteria at or after the severe sepsis onset timestamp. For example, if sepsis criteria were first satisfied at hour 10, severe sepsis criteria at hour 16, and septic shock criteria at hour 22, the admission would be sequentially labelled according to this temporal progression, with hour 22 used as the annotated septic shock onset timestamp for the admission.
- *Train–test data split*. (Fig. 1B). Following data preprocessing (Fig. 1A), MIMIC-III and AUMC were independently partitioned into 80% training, 5% validation, and 15% test splits prior to subsequent feature standardisation and missing data imputation procedures. For model development, the training and validation partitions were collectively treated as the development dataset. The same predefined training, validation, and test partitions were consistently maintained across all settings. Internal evaluation assessed model performance using held out test data from the same cohort used for model development. External evaluation assessed cross cohort generalisability by training the model on one cohort and evaluating on the test split of the other cohort. For pooled evaluation, the preprocessed development datasets from MIMIC-III and AUMC were aggregated into a unified development dataset to evaluate model performance across multi-site data distribution. Test datasets were reserved exclusively for final assessment of model performance. For pooled model development, only the training partitions from both cohorts were combined, while testpartitions remained isolated for independent evaluation. This design prevented information leakagebetween cohorts during model training and performance assessment. Detailed dataset split, cohortallocations, and admission count for evaluation setting are summarised in Table 1.

**Table 1:** Dataset split configurations and admission count across internal, external, and pooled evaluation settings.

Evaluation setting	Train set (admissions)	Validation set (admissions)	Test set (admissions)
Internal (MIMIC-III)	MIMIC-III: 40,248	MIMIC-III: 2,517	MIMIC-III: 7,546
Internal (AUMC)	AUMC: 17,902	AUMC: 1,119	AUMC: 3,358
External (MIMIC-III → AUMC)	MIMIC-III: 40,248	MIMIC-III: 2,517	AUMC: 3,358
External (AUMC → MIMIC-III)	AUMC: 17,902	AUMC: 1,119	MIMIC-III: 7,546
Pooled (Test on MIMIC-III)	MIMIC-III + AUMC: 58,150	MIMIC-III + AUMC: 3,636	MIMIC-III: 7,546
Pooled (Test on AUMC)	MIMIC-III + AUMC: 58,150	MIMIC-III + AUMC: 3,636	AUMC: 3,358
Pooled (Combined test set)	MIMIC-III + AUMC: 58,150	MIMIC-III + AUMC: 3,636	MIMIC-III + AUMC: 10,904

- *Standardisation*. (Fig. 1C). Each non-binary feature is standardised as:
  $$z=\frac{x-\mu }{\sigma }$$
  where *z* is the standardised value, *x* is the original feature value, *µ* is the mean, and *σ* is the standard deviation. Following train-test data split, feature wise mean and standard deviation values were computed independently from the MIMIC-III and AUMC development datasets and subsequently applied to their corresponding validation and test datasets during internal evaluation. For external and pooled evaluations, each cohort consistently reused its own standardisation and missing data imputation parameters established during the corresponding internal evaluation without recompilation. For example, the AUMC test set preprocessing parameters derived during internal evaluation (AUMC → AUMC) were subsequently reused during external (MIMIC-III → AUMC) and pooled evaluations. Similarly, the MIMIC-III test set preprocessing parameters derived during internal evaluation (MIMIC-III → MIMIC-III) were reused across external (AUMC → MIMIC-III) and pooled evaluations. For pooled model development, MIMIC-III and AUMC development datasets were aggregated after cohort specific preprocessing without recomputing pooled population statistics, thereby preserving cohort specific feature distributions for subsequent missing data imputation.

- *Missing data imputation* (Fig. 1D). Missing values were imputed in two stages. First, Zero Order Hold (ZOH) was applied, whereby the most recent previously observed value was carried forward ^25^. This approach preserves temporal continuity by assuming that previously recorded measurement remains informative until a newer observation becomes available. Second, any remaining missing values without a prior observation were replaced using the corresponding population mean computed from the training split.

### Model development

#### Long short-term memory (LSTM) networks

LSTM networks are a recurrent neural network (RNN) architecture designed to address the vanishing gradient problem in sequential data modelling ^26^. Each LSTM unit comprises input, output, and forget gates, which regulate information flow through the hidden state and the memory cell state. This architecture enables effective modelling of long-term temporal dependencies.

#### LiSep LSTM model architecture

The LiSep LSTM model used in this study follows the architecture introduced by Fagerström et al. for early septic shock detection. We implemented this previously validated design, consisting of four LSTM layers with 100 hidden units each, trained with dropout regularisation to reduce overfitting. The network was trained for 1,000 epochs with 40 mini-batches of 50 samples per epoch, and class imbalance was addressed by weighting positive cases three times higher than negatives. Leveraging this established architecture ensures comparability with prior work and allows our study to focus on evaluating model transferability and generalisability across cohorts rather than introducing a new model design.

#### Model training

Models were trained on three dataset compositions: MIMIC-III, AUMC, and the pooled dataset. The training data from the dataset, with each admission labelled as positive or negative for septic shock during that stay. AUROC was used to assess the discrimination performance of the model across the evaluation settings described in Table 1. Confidence intervals (95% CI) were obtained by bootstrapping across the six cross validation folds.

Six‑fold cross‑validation was used to match the training strategy described for the original LiSep LSTM model by Fagerström et al.^17^, ensuring methodological consistency and enabling direct comparison with their reported results. This configuration also maintained adequate representation of septic shock cases within each fold while balancing computational feasibility during repeated LSTM training. Positive and negative samples were first shuffled separately to preserve class balance, after which each class was divided into six folds. For each iteration, five folds were used for training and one fold for testing, ensuring that every fold served once as a test set. A separate 5% holdout validation set was used to monitor training progress and prevent overfitting. This approach produced six independently trained model instances for each dataset composition—MIMIC-III, AUMC, and the pooled dataset, allowing robust estimation of performance metrics and confidence intervals.

### Model performance evaluation

The model generates predictions at hourly intervals for each admission. Septic shock onset was defined as the first annotated hourly timestep satisfying the septic shock criteria, and model performance was assessed by comparing hourly predictions against the corresponding hourly onset labels described in “Data preprocessing” section during model development and evaluation.

**Fig. 2:**
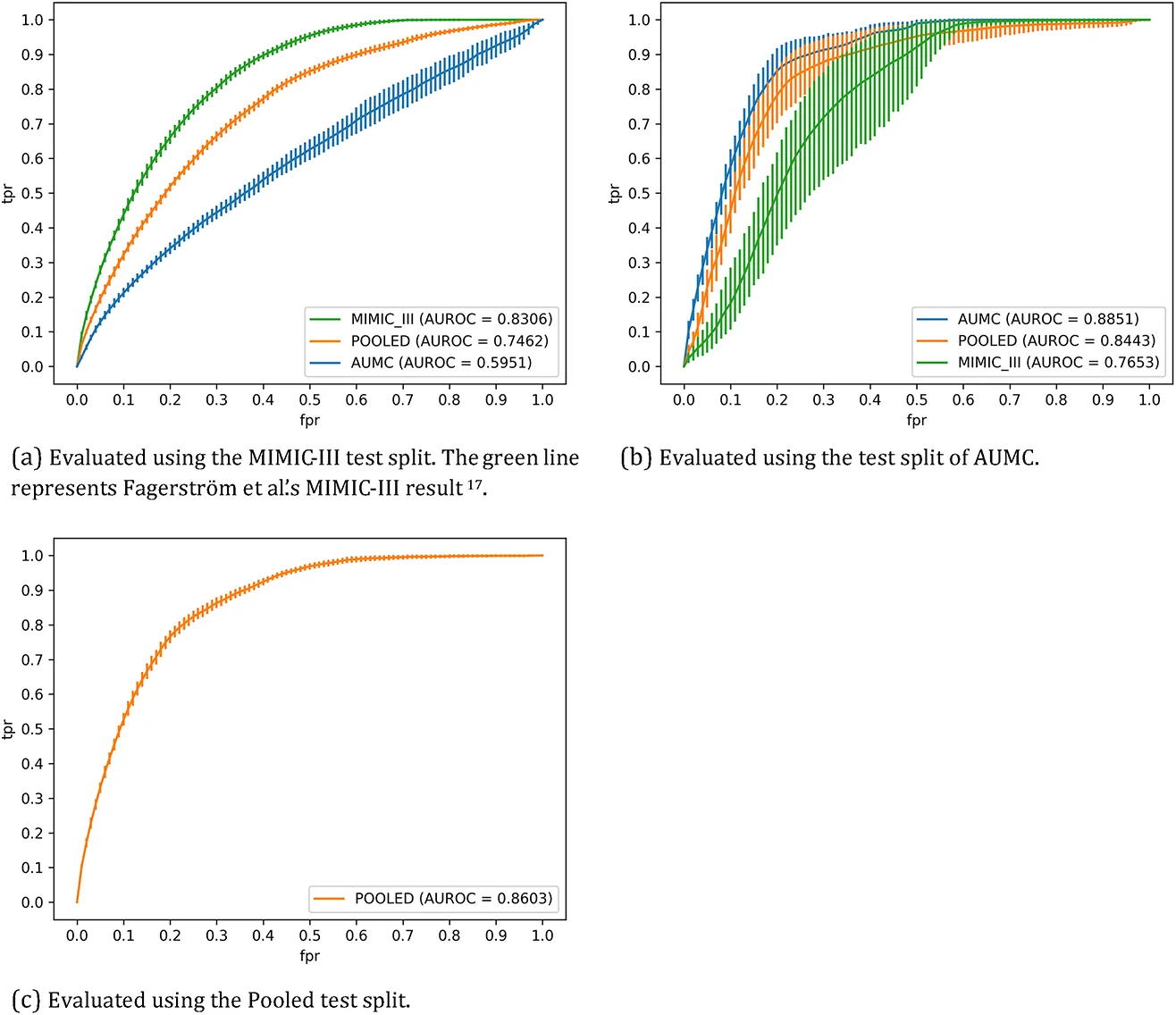
AUROC for the LiSep LSTM model, trained on different datasets or under various evaluation settings (Table 1). Error bars represent the 95% confidence interval (CI), computed by bootstrapping the evaluation results for the six independently trained model instances obtained from six-fold cross validation.

**Fig. 3:**
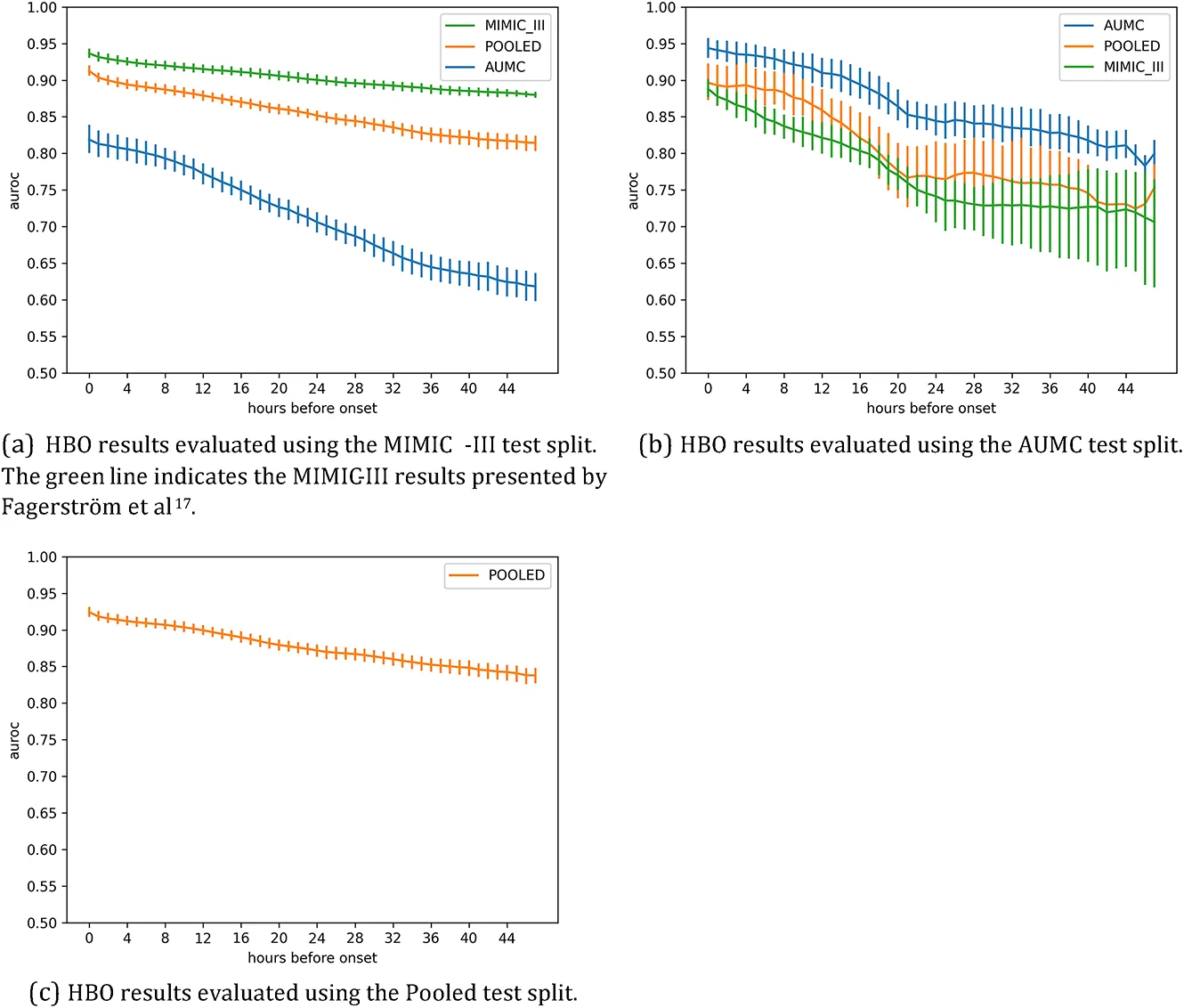
AUROC over the 48 hours in advance to septic shock onset. It compares the prediction made by the LiSep LSTM model, trained on different training datasets or under various evaluation settings. Error bars represent the 95% confidence intervals, computed by bootstrapping the evaluation results for the six independently trained model instances obtained from six-fold cross validation.

Model performance was evaluated using AUROC, HBO, Brier score, accuracy, precision, and recall. AUROC was first calculated across all hourly predictions throughout the full admission duration (Fig. 2). AUROC was subsequently computed at each hourly prediction within the 48 hours preceding septic shock onset to evaluate how predictive performance changed as onset approached (Fig. 3). HBO represents the lead time between the first positive prediction and septic shock onset for correctly identified septic shock positive admissions. As HBO is based on the earliest positive prediction, very large HBO values may reflect prolonged periods of elevated predicted risk rather than sustained clinically actionable alarms. Brier score, accuracy, precision, and recall were computed using model predictions aggregated across each admission. To provide additional context regarding the evaluation data, the total number of evaluated hourly screening instances and the corresponding positive rates within each test set are reported in Table 3.

Brier score was used to assess the accuracy and calibration of probabilistic predictions. It is defined as the mean squared difference between predicted probabilities and observed outcomes:

$$Brier Score=\frac{1}{N}{\sum}_{i=1}^{N}{\left({\mathrm{p}}_{\mathrm{i}}-{y}_{i}\right)}^{2}$$

where ${p}_{i}$ is the predicted probability at each admission stay *i*, ${y}_{i}$ is the true binary outcome (0 = no septic shock, 1 = septic shock), and *N* is the total number observations across all admissions. The score ranges from 0 to 1, with lower values indicating better calibration and more reliable probability estimates.

To complement probability-based evaluation, we also computed classification metrics by converting predicted probabilities into binary outcomes. Accuracy was defined as the proportion of correct predictions (true positives and true negatives) across all cases. Although widely used, accuracy can be misleading in imbalanced datasets where one class dominates. To provide a clearer view of model performance on the positive class, we additionally reported precision and recall. Precision represents the proportion of correctly identified positive cases among all predicted positives, reflecting the false positive burden. Low precision means many false alarms, which can cause “alert fatigue” and unnecessary interventions. High precision reassures clinicians that when the model raises an alarm, it is likely to be meaningful.

$$Precision=\frac{True Positives}{True Positives+False Positive}$$

Recall also known as sensitivity measures the proportion of true positives correctly identified among all actual positive cases, answering the clinical question: “Of all patients who developed septic shock, how many were detected by the model?”. High recall is critical in sepsis care, as missing a patient at risk of septic shock could have severe or fatal consequences.

$$Recall=\frac{True Positives}{True Positives+False Negatives}$$

To compute binary classification metrics such as accuracy, precision, and recall, predicted probabilities were converted into class labels using a decision threshold for evaluation purposes. The threshold was selected using Youden’s J statistic, defined as:

*J=Sensitivity+Specificity-1*

For each evaluation setting, Youden’s J statistic was derived from the AUROC curve, and the threshold that maximised J was selected. This approach provides a balanced trade-off between sensitivity (true positive rate) and specificity (true negative rate), identifying the operating point at which the model most effectively distinguishes between septic shock and non-septic shock cases.

### Permutation feature importance (PFI)

Understanding which features most influence the LiSep LSTM model helps interpret predictions and assess robustness across datasets. PFI provides a model-agnostic approach by quantifying how much predictive performance deteriorates when the relationship between a given feature and the outcome is disrupted ^27,28^. A large drop in AUROC after permutation indicates high relevance, whereas little or no change suggests low The procedures summarised in Algorithm 1 estimates feature relevance by comparing baseline AUROC with AUROC after permuting each feature column in turn. Permutation importance quantifies the contribution of each input feature to model discrimination. A trained LiSep LSTM model $\widehat{f}$ was evaluated using AUROC as the performance metric. For each feature *j*, the corresponding column of the feature matrix $X\in {\mathbb{R}}^{n\times p}$ was randomly permuted, breaking the association between that feature and the outcome $y\in {\mathbb{R}}^{n}$. The drop in AUROC relative to the baseline $F{I}_{j}$ = $AURO{C}_{orig}$− $AURO{C}_{perm,j}$ defines the importance of feature *j*. Features are ranked in descending order of $F{I}_{j}$, where larger values indicate greater importance for model performance.

**Algorithm 1:**
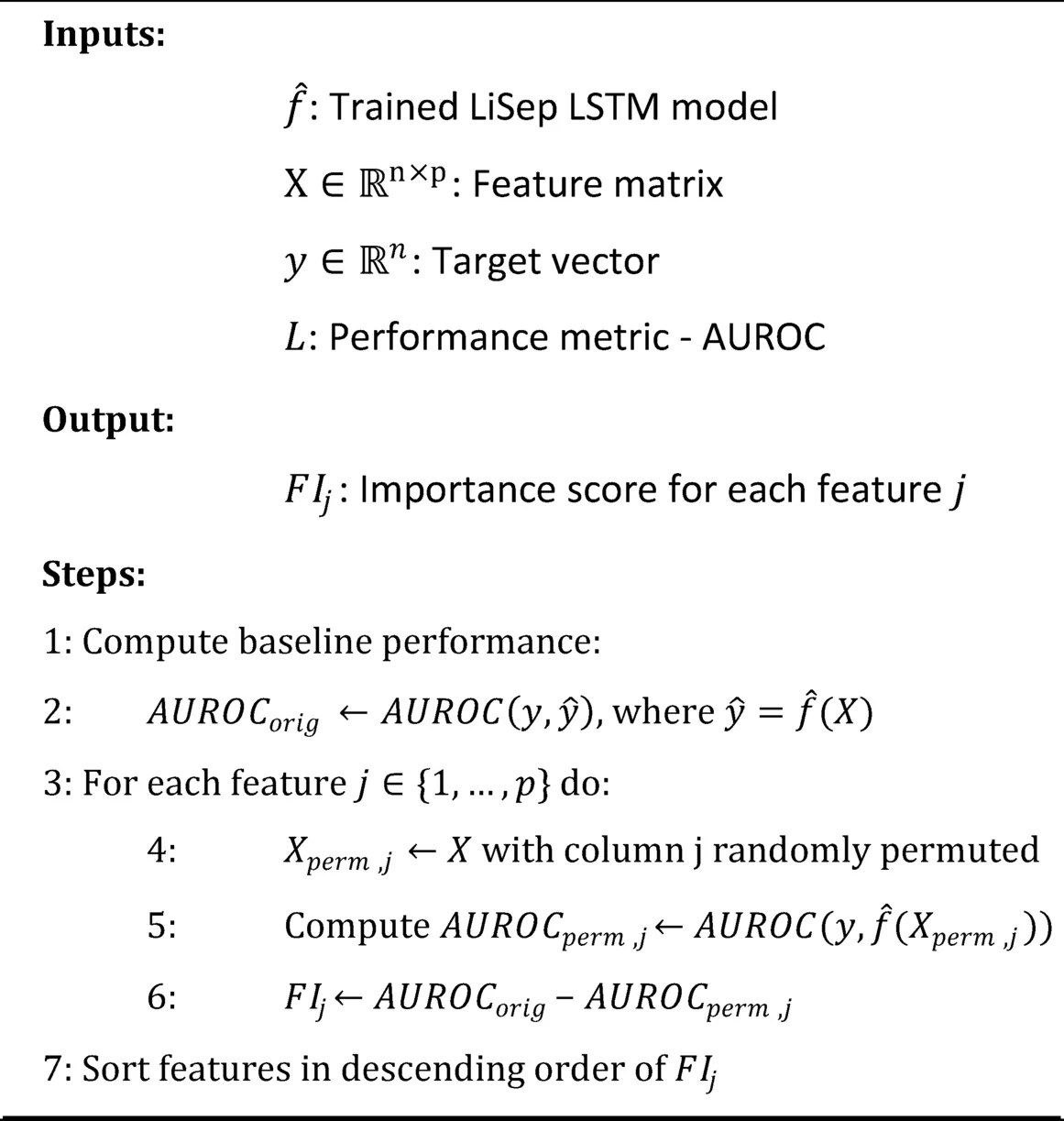
Steps for computing PFI for the LiSep LSTM model.

### Distribution shifts

**Fig. 4:**
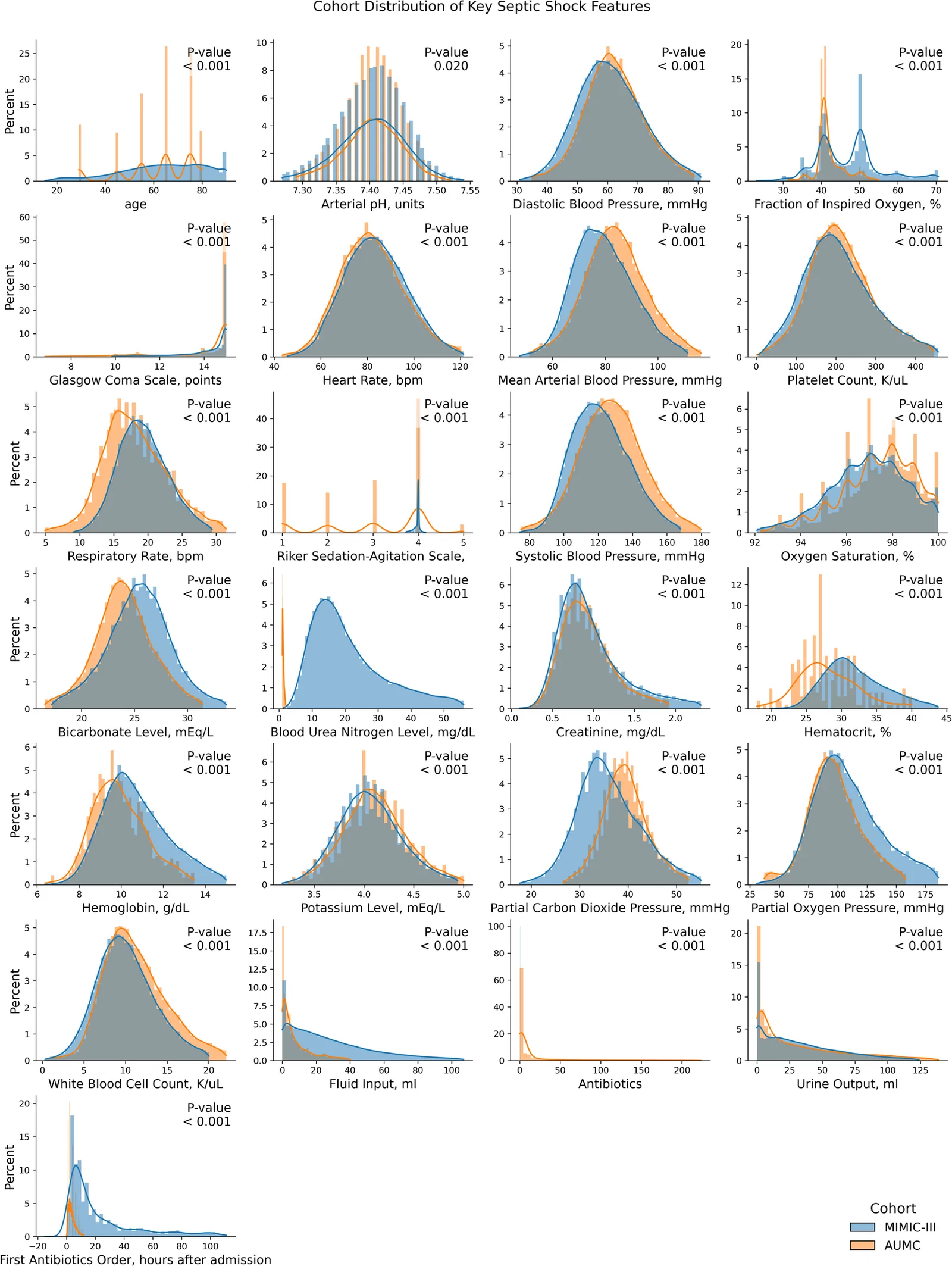
Feature distributions were averaged over time and aggregated per patient to assess cohort-level shifts between MIMIC-III and AUMC. P values from the Mann–Whitney test are shown in the top-right corner of each subplot. Distributions were normalised to the total number of admissions in each cohort.

We assessed the impact of dataset-specific distributional shifts on the performance and generalisability of the LiSep LSTM model by comparing key sepsis related feature distributions between MIMIC-III and AUMC (Fig. 4). This comparison highlighted variables most relevant for early sepsis detection and allowed us to identify cohort-specific patterns as well as systematic differences across datasets. Recognising these discrepancies provided important context for the generalisation gap observed when transferring models between cohorts. To address computational constraints and maintain sufficient resolution in the histograms, each feature was averaged across the time sequence and aggregated at the patient level. Consequently, ordinal clinical scores such as the Glasgow Coma Scale and Riker Sedation Agitation Scale may appear as non-integer values because they were derived from measurements averaged across the time sequence and aggregated at the patient level.

The original time varying antibiotics exposure feature used in the LiSep LSTM framework was retained as a predictive variable to preserve consistency with the original implementation by Fagerström et al. ^17^. In contrast, the derived descriptive variable “First Antibiotics Order”, representing elapsed time from ICU admission to first recorded antibiotic administration, was used exclusively for cohort comparison and excluded from model training and inference. To minimise potential information leakage, only observations available up to each prediction timestep were accessible to the model during training and evaluation. Furthermore, timestamps were represented relative to ICU admission and normalised to hourly resolution without retaining absolute datetime information, thereby reducing the risk of direct temporal leakage from future clinical events.

We did not evaluate models trained solely on MIMIC-III or AUMC against the pooled test set, as this configuration would not provide a meaningful assessment of generalisability. Instead, pooled evaluation was designed specifically to measure generalisation when both cohorts contribute to training and validation, thereby enabling a fair comparison with internal and external settings without violating data independence.

## Results

The LiSep LSTM model was developed to detect early onset of septic shock for each hospital admission. After preprocessing data from MIMIC-III and AUMC, the model was trained on each dataset individually and on a pooled dataset to assess performance on a test split. Input features, following Fagerström et al.^17^ and adhering to Henry et al.^7^, included demographic data, vital parameters, and hourly laboratory results during admission.

We reported the evaluation performance summary for each setting on different test sets, as shown in Table 2, which includes results by Fagerström et al. ^17^. AUROC and HBO are grouped by identical MIMIC-III and AUMC test splits, presented in Fig. 2 and 3. This includes the results presented by Fagerström et al. in Fig. 2a and 3a.

**Table 2:** Model performance of the LiSep LSTM model across internal, external, and pooled evaluations using MIMIC-III and AUMC datasets. Internal refers to training and testing within the same datasets, external refers to training on one dataset and testing on the other, and Pooled refers to training on a combined dataset.

Evaluation setting	Train set	Test set	AUROC(95% CI)	HBO Median (IQR)	Brier Score (95% CI)	Accuracy (95% CI)	Precision (95% CI)	Recall (95% CI)	Threshold
Internal	MIMIC-III	MIMIC-III	0.83 (0.82, 0.84)*	48.0 (20.0, 135.0)*	0.0939 (0.0886, 0.0992)	0.6517 (0.6174, 0.686)	**0.1724 (0.1579, 0.1869)**	0.7703 (0.7406, 0.8)	0.274
Pooled	Pooled	MIMIC-III	0.7462 (0.7389, 0.7535)	35.0 (16.0, 109.0)	0.0967 (0.0933, 0.1)	0.6224 (0.5832, 0.6616)	0.1607 (0.1479, 0.1735)	0.7715 (0.7281, 0.8148)	0.2481
External	AUMC	MIMIC-III	0.5951 (0.5651, 0.6251)	35.0 (16.0, 109.0)	0.0874 (0.0833, 0.0915)	0.6656 (0.532, 0.7993)	0.1287 (0.12, 0.1374)	0.4672 (0.2814, 0.6531)	0.0015
Internal	AUMC	AUMC	**0.8851 (0.8612, 0.909)**	47.0 (16.0, 255.0)	**0.0058 (0.0045, 0.007)**	0.761 (0.6791, 0.8428)	0.0211 (0.0167, 0.0255)	**0.9072 (0.8375, 0.9769)**	0.0125
Pooled	Pooled	AUMC	0.8443 (0.7922, 0.8963)	47.0 (16.0, 255.0)	0.0069 (0.056, 0.0083)	**0.7742 (0.7633, 0.7852)**	0.0195 (0.0163, 0.0228)	0.8273 (0.7185, 0.9362)	0.0375
External	MIMIC-III	AUMC	0.7653 (0.6817, 0.8489)	46.0 (16.0, 255.0)	0.1459 (0.0899, 0.2018)	0.5995 (0.5016, 0.6975)	0.0126 (0.0093, 0.016)	0.9047 (0.8487, 0.9607)	0.4318
Pooled	Pooled	Pooled	0.8603 (0.8509, 0.8697)	35.0 (16.0, 113.0)	0.0472 (0.0455, 0.0488)	0.7602 (0.743, 0.7774)	0.131 (0.1211, 0.1409)	0.8189 (0.7981, 0.8397)	0.168

Results are reported as mean values with 95% CI where applicable. *Result presented by Fagerström et al.^17^.

Figure 3a shows the results of the model trained on MIMIC-III, AUMC, and pooled data, assessing its performance on the test split of MIMIC-III. A similar approach was applied to the test split of AUMC, as shown in Fig. 3b. Figure 3c presents the pooled evaluation results. This setup demonstrates how exposure to heterogeneous data influences model performance and generalisation across sites, showing improved AUROC and Brier score relative to external evaluations while maintaining comparable HBO performance as shown in Table 2.

Figure 3a and b show the AUROC over 48 hours at an hourly resolution, computed by the LiSep LSTM model previously trained on the MIMIC-III, AUMC, and pooled datasets. Its performance was assessed on the MIMIC-III and AUMC test sets, respectively. The AUROCs increase as the onset of septic shock draws closer. Figure 3c presents the corresponding temporal results for the pooled evaluation, illustrating how training on combined data maintains stable AUROC trends across the 48-hour prediction window. The implications of the results in Fig. 2 and 3 will be further elaborated in the discussion section.

Table 2 presents the model performance of the LiSep LSTM across internal, external, and pooled evaluations. Internal validation produced the strongest overall performance, with the highest AUROC and lowest Brier score, demonstrating strong discrimination and well calibrated probabilities when tested on the same cohort. External validation consistently showed lower AUROC and higher Brier score, along with shorter HBO lead times, indicating poorer discrimination and less reliable probability estimates when transferring across cohorts. Pooled evaluation with MIMIC-III as the test set further improved AUROC, preserved HBO, and enhanced both precision and recall, whereas pooled evaluation with AUMC as the test set improved AUROC and accuracy while retaining HBO but did not increase precision and recall. The pooled-to-pooled evaluation further demonstrated stable performance, with AUROC and Brier score approaching internal validation, reflecting the model’s ability to maintain discrimination and temporal sensitivity when both training and testing on combined datasets.

Accuracy was moderate across all evaluations, but its clinical value was limited by class imbalance, since correct predictions were dominated by the majority class. Precision remained low across all evaluations, underscoring the persistence of false alarms, while recall only improved in pooled evaluation with MIMIC-III as the test set. Table 3 summarises the hourly screening instances and positive rates within each test set. The positive rate was 8.82% in MIMIC-III, 0.55% in AUMC, and 4.26% in the pooled set. These differences reflect variation in the frequency of positive screening labels across test sets and help contextualise the observed precision values.

**Table 3:** Summary of hourly screening instances and positive rates within the evaluated test cohorts. Positive rate represents the percentage of positive hourly screening labels among all evaluated hourly screening instances within each test cohort.

Test set	Hourly screening instances	Positive rate (%)
MIMIC-III	1,370,756	8.82
AUMC	1,686,867	0.55
Pooled	3,057,622	4.26

Cohort characteristics between MIMIC-III and AUMC demonstrated notable differences across several clinical features relevant to septic shock (Fig. 4). Histogram analyses indicated that certain laboratory variables such as blood urea nitrogen (BUN), hematocrit, and hemoglobin were sparsely recorded in AUMC, resulting in substantial missingness compared with the more consistently available measurements in MIMIC-III. Demographic feature, including age, was reported as categorical groups in AUMC, producing stepwise distributions, whereas MIMIC-III captured these variables on a continuous scale. In contrast, most vital signs, including HR, RR, blood pressure, and temperature, exhibited broadly overlapping distributions between cohorts, although wider dynamic ranges were observed in MIMIC-III, reflecting its larger sample size.

The antibiotics variable in MIMIC-III contains heterogeneous units of measurement, with most entries recorded as doses and the remainder as volumes. From this, we derived the “First Antibiotics Order” variable, representing the elapsed time in hours from ICU admission to first recorded antibiotic administration. This variable was used solely for descriptive cohort comparison and was excluded from model training and inference. While most patients in both cohorts received antibiotics within hours of admission, the timing distributions differed significantly. MIMIC-III showed a broader, right-skewed distribution with more cases of delayed initiation, whereas AUMC displayed a narrower distribution with fewer delayed administrations. Interestingly, some MIMIC-III patients were recorded as receiving antibiotics before ICU admission, suggesting treatment began in the emergency department or hospital wards prior to ICU transfer.

Across all PFI experiments, the results are shown in Supplementary Fig. 2 – Fig. 8. Internal evaluations (Supplementary Figs. 2 and 5) demonstrated the highest AUROC and calibration when evaluated on the same site as they were trained. Although the top five predictive features varied between sites, their consistent and frequent measurement within each hospital likely contributed to elevated internal performance, suggesting that reliance on site-specific lab practices may lead to an overestimation of generalisability.

External validation or cross-hospital validation, AUROC decreased and Brier score worsened across hospitals. The predictive value of lab-driven features diminished or reversed, while stable vital signs—Glasgow Coma Scale (GCS), RR, HR, and oxygen saturation (SpO₂) partially compensated. This drop reflects the instability of lab feature importance, indicating that features dominant in one hospital may be less informative in another, increasing Brier score.

Pooled training improved performance when the pooled model was evaluated on each cohort, compared with the lower performance observed when a single source model was evaluated on a different cohort, as shown in Table 2. The PFI results in Supplementary Figs. 3, 6, and 8 showed a broader and more balanced distribution of feature relevance, indicating reduced dependence on cohort-specific variables and greater model generalisability. The model increasingly relied on core physiological features, notably GCS, RR, HR, SpO₂FiO₂, PaO₂, PaCO₂, mean arterial blood pressure (MAP), while laboratory and diagnostic variables became less influential. The antibiotics feature gained higher importance in pooled-AUMC and pooled-pooled settings, reflecting the model’s ability to capture treatment timing patterns related to infection onset and intervention. Diagnoses features contributed minimally, highlighting that temporal physiological dynamics rather than baseline characteristics drive septic shock prediction. Overall, pooled training enhanced cross hospital robustness by emphasising site independent physiological and therapeutic signals that remain informative across heterogeneous ICU populations.

## Discussion

In this study, we externally validated the previously developed LiSep LSTM model using the AmsterdamUMC cohort and evaluated its generalisability across independent ICU datasets. While the model achieved strong performance under internal validation, performance declined during external validation, highlighting the impact of dataset-specific distributional shifts on model transferability. Pooled training improved overall robustness across cohorts, suggesting that incorporating more diverse training data may enhance generalisability. These findings demonstrate both the potential and the challenges of deploying deep learning models for early septic shock prediction across heterogeneous healthcare systems.

The number of septic shock patients in AUMC represents only about 10% of the size of MIMIC-III (see Supplementary Table 1). The histograms of HBO at septic shock onset (Supplementary Fig. 1) suggest that both datasets share broadly similar distributional shapes, indicating comparable trends despite differences in scale, range, or central tendency. While the smaller sample size of AUMC likely contributes to some variability in performance, the primary factor underlying reduced external validation performance is the distributional shift between cohorts. Importantly, pooled training improved performance, as reflected in Fig. 2 - 3, highlighting the benefits of leveraging larger and more diverse data sources. Importantly, HBO should be interpreted cautiously as a measure of early warning lead time. Because HBO is based on the first positive prediction within an admission, very large HBO values may reflect prolonged periods of elevated predicted risk or isolated early model activation rather than sustained clinically actionable alerts. In this study, HBO was used as a descriptive temporal performance metric rather than an optimisation target during model training. Consequently, HBO should be interpreted alongside complementary metrics such as AUROC, precision, recall, and Brier score when assessing overall model utility.

The comparative results in Table 2 highlight both the potential and the challenges of cross cohort application of the LiSep LSTM. The benefits of pooled training varied between datasets. When testing on MIMIC-III, pooled training improved both recall and AUROC, indicating greater sensitivity to septic shock cases. However, when testing on AUMC, pooled training enhanced AUROC, accuracy, and Brier score without increasing recall. This suggests that recall gains are not guaranteed when transferring to both continents. This asymmetry likely reflects differences in data representation and completeness across the two databases, such as grouped demographics and missing laboratory variables in AUMC.

From a clinical perspective, the findings emphasise that while pooled training enhances generalisability, site specific data characteristics continue to play a decisive role in determining whether recall and therefore early detection can be reliably achieved. High recall is especially beneficial in this context, as it allows the model to identify a broad pool of at risk patients, ensuring that very few septic shock cases are missed. This is crucial in a clinical setting where timely recognition and intervention can significantly improve survival. At the same time, precision remained low across all evaluation settings, suggesting a persistent burden of false alarms, which may contribute to alert fatigue and limit the model’s practical adoption without additional filtering or prioritisation strategies.

The absence of several laboratory variables in AUMC reduces the informative signal available to the model, contributing to poorer performance in external validation. Research have shown that increased missingness notably reduces predictive performance in ML models applied to healthcare data ^29,30^. Additionally, the two databases use different clinical scoring conventions, particularly in sedation assessment scales, which creates heterogeneity in feature representation. These differences in data completeness and scoring systems hinder direct model transferability, as they alter the effective feature space available during both training and evaluation. This effect is evident in Fig. 4, where external evaluations exhibit lower AUROC and Brier score compared to internal and pooled settings, illustrating how missing or inconsistently represented features weaken cross-site performance.

Distributional differences were particularly evident for Blood Urea Nitrogen, which exhibited substantially higher missingness in AUMC (86.5%) than in MIMIC-III (0.5%) (Supplementary Table 2). This discrepancy may reflect differences in laboratory measurement practices and data availability between institutions, contributing to the observed distributional shift. In contrast, the Riker Sedation Agitation Scale exhibited comparable missingness between cohorts but retained noticeable differences in distribution. As this variable is based on clinician assessment and documentation, variations in local clinical practice and recording behaviour across healthcare systems may also contribute to the observed cohort differences shown in Fig. 4.

MIMIC-III predominantly records variables as continuous with wide dynamic ranges (e.g. age), enabling the model to learn fine-grained variability. In contrast, AUMC features are frequently grouped or discretised, reflecting differences in documentation practices. This grouping is a deliberate privacy preserving measure aimed at reducing the granularity of age and weight data to minimise reidentification risks. While this enhances data security, it also reduces fine grained variability in these clinically relevant features, limiting the model’s discriminatory capacity when the model trained on MIMIC-III and evaluated by AUMC. Consequently, models trained on MIMIC-III encounter a narrower feature space when applied to AUMC, resulting in reduced discriminatory performance. Conversely, models trained on AUMC generalise better to MIMIC-III, likely because continuous data representations can better accommodate discretised patterns. Grouping continuous clinical variables into categories creates artificial boundaries that do not reflect actual physiological changes. This makes feature distributions between hospitals look more different than they are, which amplifies distributional shift.

The feature importance analysis provides insight into how cohort characteristics shape model behaviour. Among the laboratory testing variables, hemoglobin stood out with noticeably higher importance in the MIMIC-III related test site as shown in Supplementary Fig. 2–4, and 8. This reflects that hemoglobin carries measurable predictive information within MIMIC-III, even though most other laboratory variables showed limited importance. In contrast, temporal physiological features including respiratory rate and heart rate, remained influential across most evaluations. GCS showed strong importance in the MIMIC-III and remains informative under cross cohort evaluations, but its influence was more limited in the AUMC internal evaluation shown in Supplementary Fig. 5. This difference is consistent with the nature of GCS, which is recorded manually by nursing staff or clinicians and can therefore differ in frequency and documentation across cohorts.

Several limitations should be acknowledged in our study. First, the absence of clinician-diagnosed septic shock labels prevents comprehensive comparison between our model and traditional diagnostic methods. Second, MIMIC-III contains relatively outdated data (2001-2012)^16^ compared to the more recent MIMIC-IV (2008-2019) ^31^, which could affect clinical relevance. This limitation could be addressed by consolidating both databases to achieve better temporal coverage. Third, our study only includes cohorts from the US and Europe, limiting global transferability of our findings. Although this represents improvement over single-region studies, expanding to include other continents would enhance model transferability worldwide. Finally, our reliance on outdated SIRS-based definitions of sepsis and septic shock, necessary for consistency with prior studies and dataset availability, may limit the generalisability and direct applicability of our findings to current clinical practice, which now favours the Sepsis-3 criteria^1^.

Despite these limitations, our findings provide valuable insights into the challenges and potential of deep learning models for early septic shock prediction across diverse healthcare settings, particularly regarding the impact of dataset-specific feature distributions on model performance. Although we use admission level averages to ensure consistent cohort level comparison and to capture general patterns in distribution shift, a future extension could examine feature behaviour at specific HBO to explore more detailed temporal changes. Although the derived “First Antibiotics Order” variable was excluded from model training and used only for descriptive cohort comparison, treatment-timing variables of this kind may provide useful contextual information for future predictive modelling, as they may capture aspects of clinician response, infection severity, and treatment trajectories that are not fully reflected by physiological variables alone.

Recent studies have proposed alternative evaluation frameworks for early warning systems that more closely reflect real-time clinical deployment. For example, Hyland et al. (circEWS)^32^ and Valik et al. (SepsisFinder)^33^ incorporate defined prediction horizons and alarm management strategies, such as silencing periods following alerts, to reduce repeated alarms and simulate continuous patient monitoring. In contrast, the LiSep LSTM model reports the first positive prediction within an admission and does not explicitly model alarm silencing or repeated alerts. While this approach allows consistent comparison across datasets, it may not fully capture the practical constraints of real-time clinical use. Future work should explore integrating such evaluation strategies to better assess clinical utility and alarm behaviour.

Future work will focus on extending the LiSep LSTM framework to the MIMIC-IV database to improve temporal robustness and align with contemporary Sepsis-3 definitions. In addition, calibration and fine-tuning strategies will be explored to optimise model performance across datasets with differing clinical characteristics, including local Swedish hospital cohorts. Federated learning offers a possible direction for enabling collaborative model refinement across institutions while preserving local data custody and accommodating site specific data heterogeneity, although this was not evaluated in the present study^34,35^. A future extension could examine precision using a requirement for consecutive positive predictions, which may reduce isolated false positives and improve temporal stability while maintaining early warning. These developments aim to enhance the model’s predictive reliability and establish a foundation for real-time deployment within European clinical environments.

## Conclusion

The LiSep LSTM model, originally developed using the MIMIC-III dataset, was evaluated for its generalisability in early septic shock prediction across U.S. (MIMIC-III) and European (AUMC) cohorts. Internal validation showed strong discrimination and calibration, while external validation revealed performance declines due to domain shift. Pooled training mitigated these effects, improving AUROC and Brier score while maintaining comparable HBO performance.

Analysis of cohort-level distributions of key septic shock features revealed differences in laboratory completeness and documentation practices between datasets, contributing to cross-site variability. PFI further demonstrated that pooled training encouraged reliance on more consistent, site-independent features while reducing dependence on cohort-specific variables, supporting greater model interpretability and stability.

Overall, these results indicate that training on heterogeneous, multi-site data enhances model robustness, interpretability, and early detection capability. While high recall supports timely intervention, low precision highlights the need to manage false alarms before clinical deployment. These findings provide a foundation for adapting LiSep LSTM to new environments, such as Swedish hospitals, where prior evaluation of feature distributions, pooled or fine-tuned training, and local calibration will be essential for reliable implementation.

## Supplementary Information

Below is the link to the electronic supplementary material.

Supplementary Material 1

## Data Availability

All raw data used in this manuscript are publicly available to accredited researchers. MIMIC‑III database is de‑identified in accordance with HIPAA standards and can be accessed via PhysioNet^36^. Data from the AUMC database, which complies with both HIPAA and GDPR requirements, are available through the Amsterdam Medical Data Science website. The computer code used in this study is available at https://gitlab.com/liu9652516/lisep_lstm/-/tree/73af3618c86ff182f7afb2eb81caa44198a135d0/ under an open-source license.
