# Supplementary material for: Evaluating the generalisability of LiSep LSTM for early prediction of septic shock across US and european cohorts
Source: Sci Rep. 2026 Sep 10;16:28358. doi: 10.1038/s41598-026-63350-0 (PMC13562518; doi:10.1038/s41598-026-63350-0)
Supplement: Supplementary file 1 [file 41598_2026_63350_MOESM1_ESM.docx]

Evaluating the Generalisability of LiSep LSTM for Early

Prediction of Septic Shock Across US and European Cohorts

- Supplementary Materials

Hong K.Tan^[1],*^ , Logan Froese^2,3^, Daniel Wilhelms^4,5^, Josef Fagerström^6^,

and Michelle.S.Chew^4,7^

^1^Department of Anaesthesiology and Intensive Care Medicine, Linköping University Hospital, Linköping, 581 85, Sweden

^2^Department of Clinical Neuroscience, Karolinska Institutet, Stockholm, Sweden

^3^Department of Neurology, Karolinska University Hospital, Stockholm, Sweden

^4^Division of Drug Research, Department of Medical and Health Sciences, Faulty of Health Sciences, Linköping

University, Linköping, 581 83, Sweden

^5^Department of Emergency Medicine, Local Health Care Services in Central Östergötland, Region Östergötland,

Linköping, 581 91, Sweden

^6^Department of Computer and Information Science, Linköping University, Linköping, 581 83, Sweden

^7^Department of Perioeperative Medicine and Intensive Care, Karolinska University Hospital, Stockholm, Sweden
* corresponding e-mail: hong.tan@regionostergotland.se

Table of Contents

[1	List of abbreviation	3](#_Toc226704368)

[2	Population overview	4](#_Toc226704369)

[3	HBO histogram	4](#_Toc226704370)

[4	Clinical features used	5](#_Toc226704371)

[5	Feature importances	6](#_Toc226704372)

[6	TRIPOD Checklist: Prediction Model Development and Validation	13](#_Toc226704373)

# 1 List of abbreviation

MIMIC-III - Medical Information Mart for Intensive Care

AUMC - Amsterdam University Medical Center

ML - Machine learning

ICU - Intensive Care Units

LSTM – Long short-term memory

RNN – Recurrent neural network

AUROC - Area under the receiver operating characteristic curve

HBO – Hour before onset

PFI – Permutation feature importance

APH – Arterial pH

DBP – Diastolic blood pressure

FiO_2_ –Fraction of inspired oxygen

GCS – Glasgow coma scale

HR – Heart rate

MAP – Mean arterial blood pressure

PC – Platelet count

RR – Respiratory rate

RSAS – Riker sedation-agitation scale

SBP – Systolic blood pressure

SpO_2_ – Oxygen saturation

Bicarb – Bicarbonate level

BUN – Blood urea nitrogen level

CREA – Creatinine

HEMA – Hematocrit

HEMO – Hemoglobin

POTAS – Potassium level

PACO_2_ – Partial carbon dioxide pressure

PAO_2_ – Partial oxygen pressure

WBCC – White blood cell count

ANTI – Antibiotics

FI = Fluid intake

UO = Urine output

CHF = Chronic heart failure

CLD = Chronic liver disease

COI = Chronic organ insufficiency

HM = Hematological malignancy

IMM = Immune compromised

DM = Diabetes

MC = Metastatic carcinoma

# 2 Population overview

|  | | **AUMC** | | **MIMIC-III** | |
| --- | --- | --- | --- | --- | --- |
| **Statistic** | | **Amount** | **%** | **Amount** | **%** |
|  | Number of admissions | 23106 | 100 | 50373 | 100 |
|  | Male patients | 14735 | 63.77 | 28304 | 56.19 |
|  | Female patients | 7875 | 34.08 | 22069 | 43.81 |
|  | Unspecified gender patients | 496 | 2.15 | NA | NA |
|  | Patients with sepsis | 956 | 4.14 | 17060 | 33.87 |
|  | Patients with severe sepsis | 903 | 3.91 | 15680 | 31.13 |
|  | Patients with septic shock | 446 | 1.93 | 7948 | 15.78 |
|  | Total IHD | 2288 | 9.90 | 5783 | 11.50 |
|  | Sepsis IHD | 251 | 1.09 (26.26) | 2153 | 4.27 (19.2) |
|  | Severe sepsis IHD | 248 | 1.07 (27.46) | 2116 | 4.20 (20.9) |
|  | Septic shock IHD | 169 | 0.73 (37.89) | 1556 | 3.09 (32.5) |
|  | Unspecified 28-day mortality | 912 | 3.95 | 1883 | 3.74 |
|  | Sepsis 28-day mortality | 74 | 0.32 (7.74) | 1041 | 2.07 (6.10) |
|  | Severe sepsis 28-day mortality | 69 | 0.30 (7.64) | 959 | 1.90 (6.12) |
|  | Septic shock 28-day mortality | 27 | 0.12 (6.05) | 494 | 0.98 (6.22) |
|  | Mechanical ventilation during ICU stay | 16261 | 70.38 | 14143 | 28.08 |

Supplementary Table 1. Population overview generated by AUMC based on the criteria stated in LiSep LSTM. IHD refers to in-hospital deaths. These are patients who died in the hospital while showing symptoms of the indicated condition. It’s important to note the condition is not necessarily the cause of death. If two percentages are presented: the first one relative to total admissions, the other to the number of patients with the specified condition.

# 3 HBO histogram

(a) HBO histogram on MIMIC-III	(b) HBO histogram on AUMC

Supplementary Figure 1. Distribution of hours before onset for the first septic shock event in each positive admission for MIMIC-III and AUMC.

# 4 Clinical features used

| **Feature name** | **Unit of measurement** | **Minimum** | **Maximum** | **MIMIC-III Missingness (%)** | **AUMC Missingness (%)** |
| --- | --- | --- | --- | --- | --- |
| Age | year | 15 | 90 | 0 | 0 |
| Arterial pH | - | 6 | 8 | 41.8 | 5.7 |
| Diastolic Blood Pressure | mmHg | 10 | 120 | 2.7 | 6.4 |
| Fraction of Inspired Oxygen | % | 20 | 100 | 76.0 | 32.3 |
| Glasgow Coma Scale | points | 0 | 15 | 2.8 | 16.1 |
| Heart Rate | bpm | 10 | 320 | 2.7 | 0 |
| Mean Arterial Blood Pressure | mmHg | 20 | 150 | 27.0 | 0.1 |
| Platelet Count | K/*µ*L | 1 | 1000 | 4.5 | 0.7 |
| Respiratory Rate | bpm | 0 | 100 | 2.7 | 0.3 |
| Riker Sedation-Agitation Scale | - | 1 | 7 | 36.5 | 39.0 |
| Systolic Blood Pressure | mmHg | 30 | 300 | 2.7 | 0.1 |
| Oxygen Saturation | % | 20 | 100 | 2.7 | 0.2 |
| Bicarbonate Level | mEq/L | 5 | 40 | 0.8 | 2.8 |
| Blood Urea Nitrogen | mg/dL | 1 | 100 | 0.5 | 86.5 |
| Creatinine | mg/dL | 0.1 | 20 | 0.3 | 3.6 |
| Hematocrit | % | 1 | 50 | 0.3 | 98.9 |
| Hemoglobin | g/dL | 3 | 20 | 0.2 | 95.7 |
| Potassium | mEq/L | 1.5 | 10 | 0.4 | 1.9 |
| Partial Carbon Dioxide Pressure | mmHg | 10 | 100 | 42.2 | 2.8 |
| Partial Oxygen Pressure | mmHg | 20 | 200 | 44.4 | 3.6 |
| White Blood Cell Count | K/*µ*L | 0.1 | 100 | 0.4 | 0.8 |
| Fluid Input | mL | 0 | 50,000 | 0 | 0 |
| Antibiotics | - | - | - | 71.9 | 18.9 |
| Urine Output | mL | 0 | 10,000 | 0 | 0 |
| Chronic heart failure | - | 0 | 1 | 0 | 0 |
| Chronic liver disease | - | 0 | 1 | 0 | 0 |
| Chronic organ insufficiency | - | 0 | 1 | 0 | 0 |
| Diabetes | - | 0 | 1 | 0 | 0 |
| Hematological malignancy | - | 0 | 1 | 0 | 0 |
| HIV | - | 0 | 1 | 0 | 0 |
| Immune compromised | - | 0 | 1 | 0 | 0 |
| Metastatic carcinoma | - | 0 | 1 | 0 | 0 |

Supplementary Table 2. Ranges used for outlier filtering during data preprocessing. Continuous features are reported with their corresponding minimum and maximum thresholds. Features from Chronic heart failure through to Metastatic carcinoma are categorical (binary) features, indicating the presence or absence of the condition. Data missingness (%) is reported for each feature in both the MIMIC-III and AUMC cohorts.

# 5 Feature importances

Supplementary Figure 2. *Top 20 feature importances of the LiSep LSTM model trained and evaluated on the MIMIC-III dataset using the permutation feature importance (PFI).*

*Supplementary Figure 3. Top 20 feature importances of the LiSep LSTM model trained on the pooled dataset and evaluated on the MIMIC-III test split.*

*Supplementary Figure 4. Top 20 feature importances of the LiSep LSTM model trained on the AUMC dataset and evaluated on the MIMIC-III test split.*

*Supplementary Figure 5. Top 20 feature importances of the LiSep LSTM model trained and evaluated on the AUMC dataset.*

*Supplementary Figure 6. Top 20 feature importances of the LiSep LSTM model trained on the pooled dataset and evaluated on the AUMC test split.*

*Supplementary Figure 7. Top 20 feature importances of the LiSep LSTM model trained on the MIMIC-III dataset and evaluated on the AUMC test split.*

*Supplementary Figure 8. Top 20 feature importances of the LiSep LSTM model trained and evaluated on the pooled dataset.*

# 6 TRIPOD Checklist: Prediction Model Development and Validation

| **Section/Topic** | **Item** |  | **Checklist Item** | **Page** |
| --- | --- | --- | --- | --- |
| **Title and abstract** | | | | |
| Title | 1 | D;V | Identify the study as developing and/or validating a multivariable prediction model, the target population, and the outcome to be predicted. | 1 |
| Abstract | 2 | D;V | Provide a summary of objectives, study design, setting, participants, sample size, predictors, outcome, statistical analysis, results, and conclusions. | 1 |
| **Introduction** | | | | |
| Background and objectives | 3a | D;V | Explain the medical context (including whether diagnostic or prognostic) and rationale for developing or validating the multivariable prediction model, including references to existing models. | 1-2 |
|  | 3b | D;V | Specify the objectives, including whether the study describes the development or validation of the model or both. | 2 |
| **Methods** | | | | |
| Source of data | 4a | D;V | Describe the study design or source of data (e.g., randomized trial, cohort, or registry data), separately for the development and validation data sets, if applicable. | 2-3 |
|  | 4b | D;V | Specify the key study dates, including start of accrual; end of accrual; and, if applicable, end of follow-up. | 2-3 |
| Participants | 5a | D;V | Specify key elements of the study setting (e.g., primary care, secondary care, general population) including number and location of centres. | 2-3 |
|  | 5b | D;V | Describe eligibility criteria for participants. | 3-4 |
|  | 5c | D;V | Give details of treatments received, if relevant. | N/A |
| Outcome | 6a | D;V | Clearly define the outcome that is predicted by the prediction model, including how and when assessed. | 4-6 |
|  | 6b | D;V | Report any actions to blind assessment of the outcome to be predicted. | N/A |
| Predictors | 7a | D;V | Clearly define all predictors used in developing or validating the multivariable prediction model, including how and when they were measured. | 4-7 |
|  | 7b | D;V | Report any actions to blind assessment of predictors for the outcome and other predictors. | N/A |
| Sample size | 8 | D;V | Explain how the study size was arrived at. | 2-5 |
| Missing data | 9 | D;V | Describe how missing data were handled (e.g., complete-case analysis, single imputation, multiple imputation) with details of any imputation method. | 4-6 |
| Statistical analysis methods | 10a | D | Describe how predictors were handled in the analyses. | - |
|  | 10b | D | Specify type of model, all model-building procedures (including any predictor selection), and method for internal validation. | - |
|  | 10c | V | For validation, describe how the predictions were calculated. | 5-6 |
|  | 10d | D;V | Specify all measures used to assess model performance and, if relevant, to compare multiple models. | N/A |
|  | 10e | V | Describe any model updating (e.g., recalibration) arising from the validation, if done. | N/A |
| Risk groups | 11 | D;V | Provide details on how risk groups were created, if done. | N/A |
| Development vs. validation | 12 | V | For validation, identify any differences from the development data in setting, eligibility criteria, outcome, and predictors. | 3-7 |
| **Results** | | | | |
| Participants | 13a | D;V | Describe the flow of participants through the study, including the number of participants with and without the outcome and, if applicable, a summary of the follow-up time. A diagram may be helpful. | Page 5, Supp Table 1 |
|  | 13b | D;V | Describe the characteristics of the participants (basic demographics, clinical features, available predictors), including the number of participants with missing data for predictors and outcome. | Supp Table 1 |
|  | 13c | V | For validation, show a comparison with the development data of the distribution of important variables (demographics, predictors and outcome). | 14 |
| Model development | 14a | D | Specify the number of participants and outcome events in each analysis. | - |
|  | 14b | D | If done, report the unadjusted association between each candidate predictor and outcome. | - |
| Model specification | 15a | D | Present the full prediction model to allow predictions for individuals (i.e., all regression coefficients, and model intercept or baseline survival at a given time point). | - |
|  | 15b | D | Explain how to the use the prediction model. | - |
| Model performance | 16 | D;V | Report performance measures (with CIs) for the prediction model. | 10-13 |
| Model-updating | 17 | V | If done, report the results from any model updating (i.e., model specification, model performance). | N/A |
| **Discussion** | | | | |
| Limitations | 18 | D;V | Discuss any limitations of the study (such as nonrepresentative sample, few events per predictor, missing data). | 16 |
| Interpretation | 19a | V | For validation, discuss the results with reference to performance in the development data, and any other validation data. | 15-16 |
|  | 19b | D;V | Give an overall interpretation of the results, considering objectives, limitations, results from similar studies, and other relevant evidence. | 15-16 |
| Implications | 20 | D;V | Discuss the potential clinical use of the model and implications for future research. | 16 |
| **Other information** | | | | |
| Supplementary information | 21 | D;V | Provide information about the availability of supplementary resources, such as study protocol, Web calculator, and data sets. | 20 |
| Funding | 22 | D;V | Give the source of funding and the role of the funders for the present study. | 20 |

*Items relevant only to the development of a prediction model are denoted by D, items relating solely to a validation of a prediction model are denoted by V, and items relating to both are denoted D;V.
